# Aid to the Injured

**Published:** 1887-09

**Authors:** 

**Affiliations:** Atlanta, Ga.


### AID TO THE INJURED.

*Editors Atlanta Medical and Surgical Journal:*

It is of the most urgent necessity in the treatment of the injured that the city should give proper facilities for their transportation and immediate care. How often do we read in the daily papers of an injured person being carried into a drug-store and remaining there stretched out on the floor for several hours, or until death releases him from his sufferings. In every case of severe injury, as we know, there is shock as a primary result. This shock varies in degree from an evanescent pallor of the skin to the most profound cardaic depression. Often it demands the surgeon's attention and treatment before the injury itself. If not treated promptly and intelligently the patient may lose his life simply from heart-failure.

In the treatment of this condition we aim to secure: First, absolute rest of the body in the recumbent posture; second, freedom from all noises and conversation calculated to excite the nervous system; third, a steady and sufficient heart's action—this being promoted by the judicious administration of internal stimulants and the application of heat to the surface of the body.

At present it is very rarely possible to give an injured person such treatment, except after considerable delay. The injured man is removed to the nearest store and laid out upon the cold floor, a doctor being hastily summoned. When he arrives he finds a motley crowd gathered around the patient discussing his chances of life in audible whispers. The doctor is forced to treat his patient with these unfavorable surroundings.

Let us have an ambulance by all means. This should be provided with splints, bandages and such drugs as are used in surgical emergencies. It should have also a soft, thick bed, a hand-stretcher and four or five blankets. The ambulance could reach the scene of an accident very quickly if summoned by telephone. The physician called could always find in the ambulance proper stimulants and the necessary surgical dressings and appliances. By these means the patient could be immediately transferred to the hospital or his residence and be placed at once under conditions most favorable to his recovery.

New York has the most complete ambulance system of any city in the world. To each hospital having ambulances there is assigned one or more police districts. An ambulance can be called from any police station, elevated railroad station, engine-house, fire-box, or from any telephone. Arbitrary signals are used in calling an ambulance by telephone. A complete code of signals is by each telegraphic instrument, and is based on the same plan as the code of the fire department. By this arrangement an ambulance can be immediately called to the scene of an accident. As soon as a call is received at the hospital, the ringing of a large electric gong notifies the ambulance surgeon and driver; the horse is at once hitched to the ambulance (the drop-harness being used as in the fire department), and the ambulance is speeding to the injured man within one minute after the call was sent out.

More than a year ago the police and hospital authorities of Paris adopted the ambulance system of New York, deciding after investigation that the American method was more practical and efficient than that of any European system. At a small expense to the city we could have in Atlanta the New York system, and I have little doubt but that it would be the means of saving many lives. Respectfully,

"JUVENIS."

*Atlanta, Ga., August 25th, 1887.*

